# Low-Energy Chest Trauma in Older Adults: Prospective Study of Indications for Chest Computed Tomography

**DOI:** 10.5811/westjem.53963

**Published:** 2026-09-24

**Authors:** Cansu Kurt, Sinan Genç, Emre Öner, Yaşar Çatal, Ayça Koca, Müge Günalp

**Affiliations:** * Ankara University, School of Medicine, Department of Emergency Medicine, Ankara, Türkiye; † Kayseri City Hospital, Department of Emergency Medicine, Kayseri, Türkiye

## Abstract

**Introduction:**

Older adults frequently present to the emergency department (ED) with low-energy blunt chest trauma, and clinically important intrathoracic injuries may be under-detected during the initial evaluation. We aimed to evaluate whether bedside findings—particularly focal chest tenderness patterns (including anterior chest wall tenderness [ACWT] and lateral compression tenderness)—were associated with computed tomography (CT)-confirmed intrathoracic injury in older adults with low-energy blunt chest trauma, while also examining chest pain and early warning scores (EWS) as adjunct clinical variables.

**Methods:**

A single-center prospective study included 136 patients 65 years or older presenting within 24 hours of low-energy blunt trauma that did not meet prespecified high-energy criteria. The primary outcome was any intrathoracic pathology on chest CT during the index ED visit. Secondary analyses examined associations between CT-confirmed injury and bedside findings (including chest pain, ACWT, lateral compression tenderness, thoracic spine tenderness, visible chest skin trauma, and auscultatory abnormalities), routine clinical variables, and laboratory measures; rib fracture and disposition were treated as prespecified exploratory analyses. The National Early Warning Score and Modified Early Warning Score were evaluated as exploratory physiologic comparators. Routine laboratory testing at presentation included arterial blood gas analysis. Chest CT performed during the index ED visit served as the imaging reference standard, with intravenous contrast use determined by clinical indication. The National Emergency X-Radiography Utilization Study (NEXUS) chest criteria informed routine imaging selection. Logistic regression identified independent associations, and diagnostic performance metrics were calculated for key bedside tenderness findings.

**Results:**

For 136 studied patients, mean age was 78.9 (9.2) years, and 60.3% of patients were female. Ground-level falls accounted for 72.8% of injuries. Chest pain was reported in 19.9% of patients. Lateral compression tenderness (21.3%) and ACWT (25.0%) were the most common examination findings. On univariable analysis, chest pain and lateral compression tenderness were associated with intrathoracic pathology; in multivariable analysis, ACWT was the only independent bedside predictor of the primary outcome (adjusted odds ratio [OR] 4.18, 95% CI, 1.85–9.44; *P* < .001). For rib fracture, ACWT, chest pain, and lateral compression tenderness remained independently associated. For intrathoracic injury, ACWT had a sensitivity of 47.6% (95% CI, 32.0–63.6), specificity of 85.1% (95% CI, 76.3–91.6), positive predictive value of 58.8% (95% CI, 44.5–71.8), negative predictive value of 78.4% (95% CI, 72.9–83.1), a positive likelihood ratio of 3.2 (95% CI, 1.79–5.70), and a negative likelihood ratio of 0.62 (95% CI, 0.46–0.83), supporting its role as a rule-in bedside cue. Lateral compression tenderness was also associated with intrathoracic injury (OR 3.81, 95% CI, 1.62–8.94; *P* = .002).

**Conclusion:**

In older adults with low-energy blunt chest trauma, focal chest tenderness findings—especially anterior chest wall tenderness, lateral compression tenderness, and chest pain—were associated with higher odds of CT-confirmed thoracic injury; however, ACWT was the only independent bedside predictor of the primary outcome. These findings function as rule-in rather than rule-out cues. Early warning scores showed limited utility for anatomic injury detection in this cohort and should not be interpreted as trauma-specific imaging decision tools.

## INTRODUCTION

Older adults frequently present to the emergency department (ED) with low-energy blunt chest trauma, most commonly due to ground-level falls.^1^,^2^ Although many appear clinically stable, intrathoracic injuries—including rib fractures, small pneumothoraces or hemothoraces, and early pulmonary contusions—are often underdetected on initial radiographs.^3^,^4^ Even apparently minor injuries can carry disproportionate morbidity in the context of frailty and reduced physiological reserve.^5^–^7^ These challenges highlight the tension between avoiding missed injuries and minimizing unnecessary imaging, radiation exposure, and downstream resource use.^3^,^4^

Several decision frameworks provide structured approaches to initial imaging.^8^ However, most were developed in general adult populations and may not be calibrated to the risk profiles of older adults with low-energy mechanisms.^8^ Age-related changes in pain perception, chest wall compliance, and vital-sign responses can alter the clinical expression of injury, potentially modifying the predictive value of bedside examination findings and physiological indices in this group.^9^,^10^ Furthermore, frailty and reduced physiologic reserve may blunt symptom reporting and physiologic responses, underscoring the importance of simple, reproducible bedside signs.^5^–^7^

Among bedside cues, anterior chest wall tenderness (ACWT) is an immediately available finding that plausibly correlates with underlying thoracic injury.^9^,^10^ However, geriatric-specific evidence quantifying its diagnostic accuracy—and comparing its performance with other routine clinical data—remains limited.^9^,^10^ In parallel, early-warning scores such as the National Early Warning Score (NEWS) and Modified Early Warning Score (MEWS) were designed to detect global clinical deterioration rather than specific anatomical injury, and their utility in identifying intrathoracic injury after low-energy trauma in older adults remains uncertain.^11^,^12^

Accordingly, we aimed to evaluate whether bedside findings—particularly focal chest tenderness findings, including ACWT and lateral compression tenderness—were associated with computed tomography (CT)-confirmed intrathoracic injury in older adults with low-energy blunt chest trauma. Secondary objectives were to explore associations with rib fracture and clinical disposition. We prespecified ACWT as the primary bedside variable of interest, while also evaluating lateral compression tenderness and chest pain as additional clinically relevant findings. We examined NEWS and MEWS as exploratory physiologic variables.

## METHODS

### Study Design and Setting

We conducted this prospective, single-center study in a tertiary ED. Of patients 65 years of age and older presenting within 24 hours after low-energy blunt trauma, 136 were eligible for inclusion. (Figure). Written informed consent was obtained from all participants. The study protocol was approved by the Ankara University Ethics Committee (Decision No. i06-430-24; June 13, 2024).

**Population Health Research Capsule**

What do we already know about this issue?

*In older adults with apparently minor blunt chest trauma, clinically important thoracic injuries may be missed on initial assessment*.

What was the research question?

*Do bedside findings—especially anterior chest-wall tenderness—predict computed tomography (CT)-confirmed intrathoracic injury?*

What was the major finding of the study?

*Anterior chest wall tenderness independently predicted injury (aOR 4.18, 95% CI, 1.85–9.44; P < .001)*.

How does this improve population health?

*A simple bedside tenderness finding may help identify higher risk older adults who warrant selective chest CT and closer evaluation*.

### Participants

Consecutive adults 65 years of age and older presenting within 24 hours of low-energy blunt chest trauma were screened. Inclusion criteria included age 65 and older, blunt chest trauma from low-energy mechanisms that did not meet prespecified high-energy thresholds, and presentation within 24 hours. Exclusion criteria (*a priori*) were high-energy mechanisms (eg, motor vehicle collision ≥40 mph [64 km/hour], fall >2 m), penetrating trauma, multisystem major trauma requiring immediate operative or intensive care unit (ICU) resuscitation, transfer after prior imaging/management, inability to provide consent, or advanced cognitive impairment precluding study procedures without a surrogate. Screening logs were maintained.

### Variables and Data Collection

At triage and initial clinician assessment, we prospectively recorded demographics, comorbidities (including Charlson Comorbidity Index [CCI]), medications (antiplatelets/anticoagulants), mechanism of injury, symptoms (including chest pain and dyspnea), physical examination findings (including ACWT, lateral compression tenderness, thoracic spine tenderness, visible chest skin trauma, chest wall deformity, and auscultatory abnormalities), and vital signs. After calculating the EWSs from the first complete set of vital signs (NEWS and MEWS), they were analyzed as exploratory physiologic comparators. Routine laboratory testing at presentation included complete blood count, electrolytes, renal function, serum lactate, and arterial blood gas analysis.

Data capture followed routine clinical workflow: Treating clinicians documented variables contemporaneously at the bedside using a structured form; and research staff performed periodic completeness checks before database lock. We prespecified variable definitions and coding rules to minimize misclassification and support reproducibility. Only patients with complete data for prespecified study variables were included in the final analytic cohort; no imputation was performed for missing values.

### Imaging Strategy and Reference Standard

Imaging followed standard ED practice, informed by decision frameworks (eg, National Emergency X-Radiography Utilization Study [NEXUS] chest criteria), clinician judgment, and shared decision-making. Chest CT performed during the index visit served as the imaging reference standard for outcome ascertainment, with intravenous contrast use determined by clinical indication at the discretion of the treating team. Although NEXUS chest criteria informed routine imaging selection, it was not evaluated as a reference standard. Injury subtypes included rib fracture, pneumothorax, hemothorax, pulmonary contusion, and other clinically relevant thoracic injuries predefined in the protocol.

### Outcomes

The primary outcome was the presence of any intrathoracic pathology on chest CT during the index ED visit (pneumothorax, hemothorax, rib fracture, vascular injury, sternal fracture, diaphragmatic rupture, or pulmonary contusion). Secondary analyses examined associations between CT-confirmed intrathoracic pathology and bedside findings (history and physical examination, including chest pain, ACWT, lateral compression tenderness, thoracic spine tenderness, visible chest skin trauma, and auscultatory abnormalities), vital signs, laboratory results, and EWSs. Rib fracture and clinical disposition (ED discharge, ward admission, or ICU admission) were treated as prespecified exploratory analyses, and in hospital mortality was recorded descriptively.

### Sample Size Considerations

Given limited prior evidence, we calculated the sample size for multivariable logistic regression targeting detection of intrathoracic injury. Age was modeled as a continuous predictor, accounting for correlation among covariates (*R*^2^ ≈ 0.17). With *α* = .05, 80% power, and events-per-variable principles applied, the required sample size was *n* = 136, which was achieved.

### Statistical Analysis

We summarized continuous variables as mean (standard deviation) or median (interquartile range), depending on distribution (assessed with the Shapiro–Wilk test). We presented categorical variables as counts and percentages. Between-group comparisons used *χ*^2^ or Fisher exact tests for categorical data, Student *t* tests for normally distributed continuous data, and the Mann–Whitney *U* test for nonnormal or ordinal data. When more than two groups were compared, we applied analysis of variance or Kruskal–Wallis.

To identify independent associations, we fitted multivariable logistic regression models for the primary endpoint of any intrathoracic pathology on CT and, as a prespecified exploratory subtype analysis, for rib fractures because they were the most frequent specific injury; other individual injury subtypes were not modeled separately because event counts were insufficient for stable multivariable estimation. Candidate covariates were prespecified on clinical grounds and/or showed evidence of association in univariable analyses. Adjusted odds ratios (aOR) with 95% confidence intervals were reported. We assessed multicollinearity using variance inflation factors. Statistical significance was set at *P* < .05 (two-sided). For ACWT, we additionally calculated sensitivity, specificity, positive likelihood ratio (LR+), and negative likelihood ratio (LR−) to characterize diagnostic performance.

## RESULTS

### Cohort and Presentation

Among 136 patients, 60.3% were female, with a mean (SD) age of 78.9 (9.2) years. Approximately half of the cohort had a high comorbidity burden by CCI category. The most common comorbidities were coronary artery disease (30.9%) and diabetes mellitus (28.7%; Table 1). All injuries were low energy, most frequently due to ground-level falls (72.8%).

On examination, the most common findings were ACWT (25.0%) and lateral compression tenderness (21.3%); no chest wall deformity was observed. A complete panel of physical-examination findings—including thoracic spine tenderness, visible chest skin trauma, chest wall deformity, and auscultatory abnormalities—was documented so that focal tenderness findings could be interpreted within the context of the full examination panel. Early warning scores at presentation were predominantly low risk. The distribution of CT-confirmed intrathoracic injuries is presented in Table 2.

### Laboratory Findings and Vital Signs

Vital signs, EWSs, and arterial blood gas parameters did not materially distinguish patients with and without intrathoracic injury.

### Predictors of Intrathoracic Pathology

On univariable analysis, chest pain (OR 3.83, 95% CI, 1.60–9.19; *P* = .003), ACWT (OR 4.18, 95% CI, 1.85–9.44; *P* < .001), and lateral chest-wall compression tenderness (OR 3.81, 95% CI, 1.62–8.94; *P* = .002) were associated with CT-confirmed intrathoracic injury (Table 3). In multivariable modeling, ACWT was the only independent bedside predictor of the primary outcome. Detailed univariable odds ratios with 95% confidence intervals and *P* values are presented in Table 3.

### Predictors of Rib Fracture

For rib fracture, ACWT (OR 5.20, 95% CI, 2.27–11.92; *P* < .001), chest pain (OR 4.64, 95% CI, 1.92–11.25; *P* < .001), and lateral compression tenderness (OR 4.65, 95% CI, 1.96–11.03; *P* < .001) were associated with CT-confirmed rib fracture on univariable analysis (Table 4).

### Diagnostic Performance

For intrathoracic injury, ACWT demonstrated modest sensitivity but useful specificity and a positive likelihood ratio, supporting its role as a rule-in bedside cue rather than a stand-alone screening test. Lateral compression tenderness showed a similar directional association and may provide additional bedside support for suspicion of injury, particularly rib fracture. Thoracic spine tenderness, visible chest skin trauma, and abnormal auscultation did not discriminate injury. Detailed diagnostic performance estimates are presented in Table 5. Physical-examination findings, mechanism of injury, EWS categories, and oxygen support status are summarized in Table 6.

### Disposition and Downstream Care

Disposition reflected overall clinician judgment and downstream care needs rather than a prespecified thoracic-trauma admission rule; some patients were discharged from the ED, while others required ward admission, and a smaller subset required ICU care. In hospital mortality was rare. In adjusted analyses, serum lactate was associated with hospital admission; however, this should be interpreted cautiously because admission was not prospectively classified using prespecified thoracic-trauma criteria.

## DISCUSSION

### Principal Findings

In older adults with low-energy blunt chest trauma, ACWT was the only bedside finding independently associated with CT-confirmed intrathoracic injury and was strongly predictive of rib fracture. Sensitivity was modest, indicating that the absence of tenderness does not reliably exclude injury. However, the corresponding specificity and positive likelihood ratio support ACWT as a useful rule-in cue when clinical concern persists. Early warning scores at presentation showed limited discriminatory value for detecting intrathoracic injury. Serum lactate was associated with hospital admission; however, this association should be interpreted cautiously because admission was a heterogeneous, nonstandardized outcome.

### Comparison with Previous Studies

Prior studies in older adults with low-energy trauma have shown that bedside examination alone has limited diagnostic yield for thoracic injury, a pattern reproduced in this cohort.^9^,^10^ In a retrospective cohort of older adults with low-energy blunt trauma, Kania et al reported that physical examination findings had limited diagnostic utility for injury prediction.^10^ In contrast, our prospective design captured predefined bedside findings and evaluated their association with CT-confirmed intrathoracic injury in a focused low-energy cohort undergoing chest CT. The findings also align with evidence that CT detects injuries frequently missed on radiography, including rib fractures, small pneumothoraces, hemothoraces, and early pulmonary contusions.^3^,^4^

In a multicenter prospective cohort of 5,912 patients with blunt chest trauma who underwent both chest radiography and chest CT, Langdorf et al reported that chest CT identified occult thoracic injuries in 71% of injured patients and in approximately one-quarter of all patients with blunt chest trauma, and that more than one third of these occult injuries prompted an intervention.^13^ Although that cohort included patients of all ages and mixed mechanisms, our study focuses on older adults with low-energy trauma, a narrower clinical setting in which selective CT decisions may be particularly challenging.^13^ Within this setting, focal tenderness findings remained clinically relevant: ACWT was the only independent bedside predictor of CT-confirmed intrathoracic injury, whereas lateral compression tenderness and chest pain were also associated with injury and remained independently associated with rib fracture. By contrast, EWSs were not designed to detect anatomic injury and did not provide useful discrimination in this cohort.^11^,^12^

We did not formally compare our findings with established rib-fracture severity or thoracic trauma scoring systems, because these tools are generally used for prognostication or disposition after established injury rather than for initial prediction of CT-confirmed injury; this remains an important area for future comparative study.

### Interpretation and Clinical Implications

Frailty, polypharmacy, and cognitive impairment in this population can blunt symptom reporting and physiological responses, increasing the relevance of simple, reproducible bedside signs.^5^–^7^ In practice, focal tenderness findings—especially ACWT and lateral compression tenderness—may support a lower threshold for chest CT when interpreted alongside chest pain and the overall clinical picture. However, these findings should be treated as rule-in rather than rule-out cues, and their absence should not be used to exclude injury. In this cohort, NEWS and MEWS showed limited discriminatory value and should not be used in isolation as a trigger for chest imaging.^11^,^12^

These data support further study of geriatric-specific refinement of existing decision frameworks, particularly regarding how focal tenderness findings might be incorporated; external validation and impact evaluation are required before implementation.

### Operational Considerations

If externally validated, a selective CT approach informed by focused bedside assessment could be feasible to implement through standardized palpation for ACWT and lateral compression tenderness, consistent documentation of associated findings (including chest pain), and selective CT use when overall clinical concern remains elevated. A short checklist or electronic prompt may improve consistency of documentation, although the impact of such strategies remains untested.

### External Validity

Although conducted at a single center, the core elements of our study findings—mechanism, symptoms, focused palpation, and selective CT—are broadly applicable and likely generalizable to diverse ED settings. Case mix (eg, frailty and anticoagulant use) may alter absolute risks, but the use of concrete bedside cues to inform imaging decisions is expected to persist.

## STRENGTHS AND LIMITATIONS

Strengths include prospective enrollment, capture of a complete physical-examination panel, and use of chest CT as the reference standard. Limitations include the single-center setting, modest sample size, and limited precision for uncommon injuries. We also did not prospectively classify admissions by formal thoracic-trauma admission criteria, which limits interpretation of the disposition analysis. In addition, we did not formally compare our findings with established chest-trauma or rib-fracture scoring systems. Laboratory associations were exploratory and should not be interpreted as clinically definitive.

### Future Directions

Multicenter studies should externally validate these findings, quantify the independent contribution of ACWT, and examine geriatric-specific calibration of existing decision frameworks. Comparative evaluations of selective, cue-informed CT strategies versus usual care should address missed-injury risk, downstream use, radiation exposure, time to diagnosis, and patient-centered outcomes.

Incorporating frailty measures and targeted physiologic variables may refine future models; however, any role for lactate in disposition assessment should be considered exploratory, especially because admissions were not prospectively categorized by formal thoracic-trauma criteria. Future research should also compare focal tenderness findings with established thoracic-trauma scoring systems in older adults.

## CONCLUSION

In older adults with low-energy blunt chest trauma, focal chest tenderness findings—especially ACWT, lateral compression tenderness, and chest pain—were associated with higher odds of CT-confirmed thoracic injury; however, anterior chest wall tenderness was the only independent bedside predictor of the primary outcome. These findings function as rule-in rather than rule-out cues, and their absence does not reliably exclude injury. When clinical concern persists, selective CT remains warranted. In this setting, early warning scores were not useful for anatomic injury detection, and laboratory- and disposition-related associations should be interpreted cautiously.

## Figures and Tables

**Figure:**
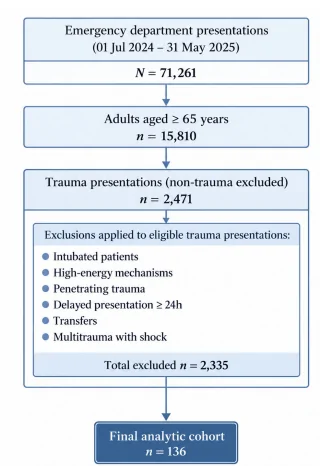
Study flow and cohort selection in a prospective single-center study of older adults (≥ 65 years) presenting to the emergency department within 24 hours of low-energy blunt chest trauma. *h*, hours.

**Table 1:** Baseline demographic, clinical, and comorbidity characteristics of a prospective cohort of older adults (≥ 65 years) presenting to the emergency department within 24 hours in a study evaluating whether bedside findings of low-energy blunt chest trauma were associated with computed tomography-confirmed intrathoracic injury.

Variable	*n* (%) or mean (SD)
Age, years	78.9 (9.2)
Sex	
Male	54 (39.7)
Female	82 (60.3)
Charlson Comorbidity Index category	
Low	11 (8.1)
Intermediate	57 (41.9)
High	68 (50.0)
Chronic medical conditions	
Chronic pulmonary disease	11 (8.1)
Coronary artery disease	42 (30.9)
Congestive heart failure	16 (11.8)
Cerebrovascular disease	14 (10.3)
Diabetes mellitus	39 (28.7)
Antiplatelet use	37 (27.2)
Anticoagulant use	40 (29.4)
Symptoms at presentation	
Chest pain	27 (19.9)
Dyspnea	15 (11.0)

Data are n (%) unless otherwise indicated. Age is presented as mean (SD).

*SD*, standard deviation.

**Table 2:** Distribution of computed tomography–confirmed intrathoracic injuries during the index emergency department visit in a prospective cohort of older adults with low-energy blunt chest trauma.

Injury	*N* = 136 *n* (%)
Pneumothorax	2 (1.4)
Hemothorax	9 (6.6)
Rib fracture	42 (30.8)
Vascular injury	0 (0)
Sternal fracture	0 (0)
Diaphragmatic rupture	0 (0)
Pulmonary confusion	12 (8.8)

Data are expressed as number (percentage) unless otherwise indicated. All injuries were confirmed by computed tomography.

**Table 3:** Univariable logistic regression analyses examining associations between patient characteristics, bedside clinical findings, vital signs, arterial blood gas parameters, and routine laboratory values and the presence of any computed tomography–confirmed intrathoracic injury in older adults with low-energy blunt chest trauma.

Variable	OR (95% CI)	*P* value
Sex (male)	1.19 (0.57–2.47)	.640
Age	1.03 (0.99–1.07)	.175
Charlson Comorbidity Index categories		.741
Low	Reference	-
Intermediate	1.23 (0.29–5.19)	.777
High	1.55 (0.38–6.38)	.544
Chest pain	3.83 (1.60–9.19)	.003*
Dyspnea	2.50 (0.84–7.38)	.098
Visible chest skin trauma	0.54 (0.11–2.71)	.453
Anterior chest wall tenderness on palpation	4.18 (1.85–9.44)	<.001*
Tenderness on compression of the lateral chest wall	3.81 (1.62–8.94)	.002*
Tenderness over the thoracic vertebrae	0.86 (0.25–2.95)	.807
Abnormal auscultation findings	2.02 (0.39–10.45)	.400
pH	0.77 (0.34–1.75)	.538
PaO_2_ (mm Hg)	0.77 (0.34–1.73)	.531
PaCO_2_ (mm. Hg)	0.73 (0.35–1.49)	.383
Lactate (mmol/L)	0.69 (0.28–1.71)	.425
Base excess (mmol/L)	0.98 (0.31–3.04)	.966

Note: Bold indicates statistical significance.

*mm Hg*, millimeters of mercury; mmol/L, millimoles per liter; *OR*, odds ratio; *pH*, potential of hydrogen; *PaO_2_*, arterial partial pressure of oxygen; *PaCO_2_*, arterial partial pressure of carbon dioxide.

**Table 4:** Univariable logistic regression analyses examining associations between patient characteristics, bedside clinical findings, physiological variables, and routine laboratory measures and computed tomography–confirmed rib fracture among older adults with low-energy blunt chest trauma.

Variable	OR (95% CI)	*P* value
Sex (male)	1.10 (0.52–2.33)	.798
Age	1.01 (0.97–1.05)	.522
Charlson Comorbidity Index categories		.920
Low	Reference	-
Intermediate	1.13 (0.27–4.80)	.865
High	1.27 (0.31–5.28)	.737
Chest pain	4.64 (1.92–11.25)	< .001*
Dyspnea	2.92 (0.98–8.70)	.053
Visible chest skin trauma	0.62 (0.12–3.13)	.564
Anterior chest-wall tenderness to palpation	5.20 (2.27–11.91)	< .001*
Tenderness on lateral compression	4.65 (1.96–11.03)	< .001*
Thoracic-spine tenderness	0.99 (0.30–3.43)	.993
Abnormal auscultation	2.33 (0.45–12.07)	.312
pH	0.64 (0.27–1.52)	.314
PaO_2_ (mm Hg)	0.91 (0.40–2.10)	.830
PaCO_2_ (mm Hg)	0.65 (0.31–1.35)	.248
Lactate (mmol/L)	0.82 (0.33–2.03)	.665
Base excess (mmol/L)	1.14 (0.36–3.55)	.828

Note: Bold indicates statistical significance.

*mm Hg*, millimeters of mercury; mmol/L, millimoles per liter; *OR*, odds ratio; *pH*, potential of hydrogen; *PaO_2_*, partial pressure of oxygen; *PaCO_2_*, partial pressure of carbon dioxide.

**Table 5:** Diagnostic performance of anterior chest-wall tenderness for computed tomography–confirmed intrathoracic injury in a prospective cohort of older adults with low-energy blunt chest trauma.

Measure	Estimate (95% CI)
Sensitivity	47.6% (32.0–63.6)
Specificity	85.1% (76.3–91.6)
Positive predictive value	58.8% (44.5–71.8)
Negative predictive value	78.4% (72.9–83.1)
Positive likelihood ratio	3.2 (1.79–5.70)
Negative likelihood ratio	0.62 (0.46–0.83)

Diagnostic performance metrics are presented with 95% confidence intervals.

**Table 6:** Physical examination findings, mechanism of injury, National Early Warning Score, Modified Early Warning Score, and oxygen support status stratified by the presence or absence of computed tomography–confirmed intrathoracic injury in a prospective cohort of older adults with low-energy blunt chest trauma.

Variable	n	Injury: Yes, *n* (%)	Injury: No, *n* (%)	*P* value
Physical examination finding				
Visible chest skin trauma	9	2 (22.2%)	7 (77.8%)	.718
Visible chest-wall deformity	0	0 (0%)	0 (0%)	-
Anterior chest-wall tenderness (on palpation)	34	20 (58.8%)	14 (41.2%)	< .001*
Lateral chest-wall compression tenderness	29	17 (58.6%)	12 (41.4%)	< .001*
Thoracic vertebral tenderness	13	4 (30.8%)	9 (69.2%)	1.0
Abnormal auscultation	6	3 (50%)	3 (50%)	.406
Mechanism of trauma				
Motor vehicle collision	11	1 (9.1%)	10 (90.9%)	
Motorcycle crash	3	1 (33.3%)	2 (66.7%)	
Pedestrian injury	2	1 (50%)	1 (50%)	
Same-level fall	99	37 (37.4%)	62 (62.6%)	
Other falls	19	5 (26.3%)	14 (73.7%)	
Blunt object injury	2	1 (50%)	1 (50%)	
NEWS score				
Low-Intermediate risk	132	46 (34.8%)	86 (65.2%)	.300
High risk	4	0 (0%)	4 (100%)	.300
MEWS score				
Low risk	136	46 (33.8%)	90 (66.2%)	NA
High risk	0	NA	NA	NA
O_2_ support	5	1 (20%)	4 (80%)	.662

*MEWS*, Modified Early Warning Score; *NEWS*, National Early Warning Score; *O_2_*, oxygen.
